# Supplementary material for: E3 ligase FBXW7 suppresses brown fat expansion and browning of white fat
Source: EMBO Rep. 2025 Jan 2;26(3):748–67. doi: 10.1038/s44319-024-00337-w (PMC11811183; doi:10.1038/s44319-024-00337-w)
Supplement: Supplementary file 1 — Appendix [file 44319_2024_337_MOESM1_ESM.pdf]

Table of Content

Appendix Fig. S1	2
Appendix Fig. S2	3
Appendix Fig. S3	4
Appendix Fig. S4	5
Appendix Fig. S5	6
Appendix Fig. S6	7
Appendix Fig. S7	8
Appendix Fig. S8	9
Appendix Fig. S9	10
Appendix Fig. S10	11
Appendix Fig. S11	12
Appendix Fig. S12	13
Appendix Fig. S13	14
Appendix Fig. S14	15
Appendix Table S1	16
Appendix Table S2	17

Appendix Figure S1

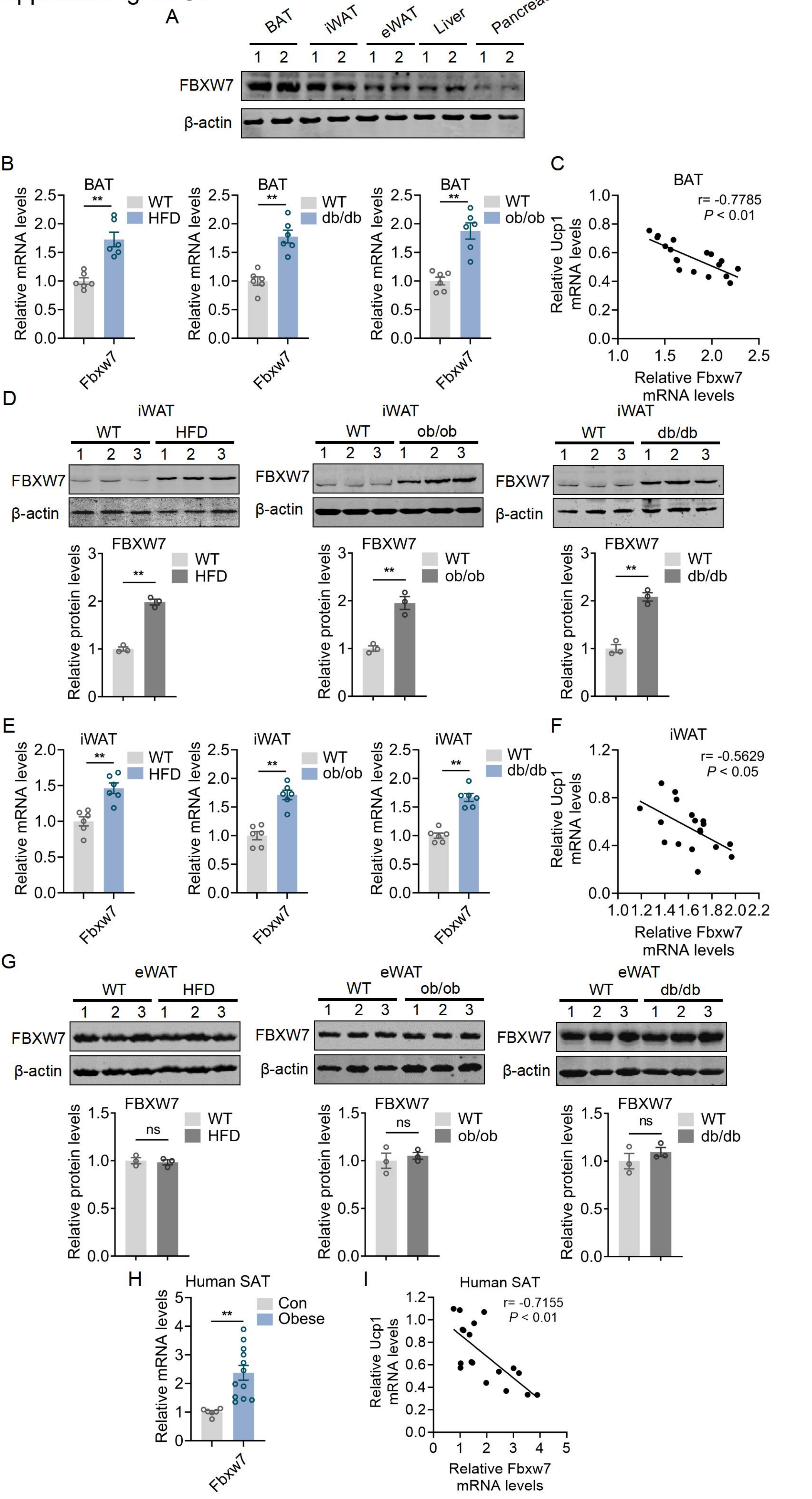

Appendix Figure S1. Expression patterns of FBXW7 in adipose tissues.

(A)Representative protein levels of FBXW7 in BAT, iWAT, eWAT, Liver and pancreas of WT mice.

(B) Relative mRNA level of Fbxw7 in BAT of HFD, ob/ob and db/db mice, n=6.

(C) Correlation of Fbxw7 and Ucp1 levels in BAT of obese mice.

(D) Representative protein levels of FBXW7 in iWAT of HFD, ob/ob and db/db mice, n=3.

(E) Relative mRNA level of Fbxw7 in iWAT of HFD, ob/ob and db/db mice, n=6.

(F) Correlation of Fbxw7 and Ucp1 levels in iWAT of obese mice.

(G) Representative protein levels of FBXW7 in eWAT of HFD, ob/ob and db/db mice, n=3.

(H) Relative mRNA level of Fbxw7 in SAT of control and obese subjects, n=6.

(I) Correlation of Fbxw7 and Ucp1 levels in SAT of obese subjects.

Data information: All data are representative of three individual experiments. n refers to biological replicates. (B, D, E, G, H) Data are presented as mean ± SEM, unpaired two-tailed Student's t-test, $^{**}p<0.01$. (C) Pearson correlation coefficients, $r=-0.7785$, $^{**}p<0.01$. (F) Pearson correlation coefficients, $r=-0.5629$, $^{*}p<0.05$. (I), Pearson correlation coefficients, $r=-0.7155$, $^{**}p<0.01$.

Appendix Figure S2

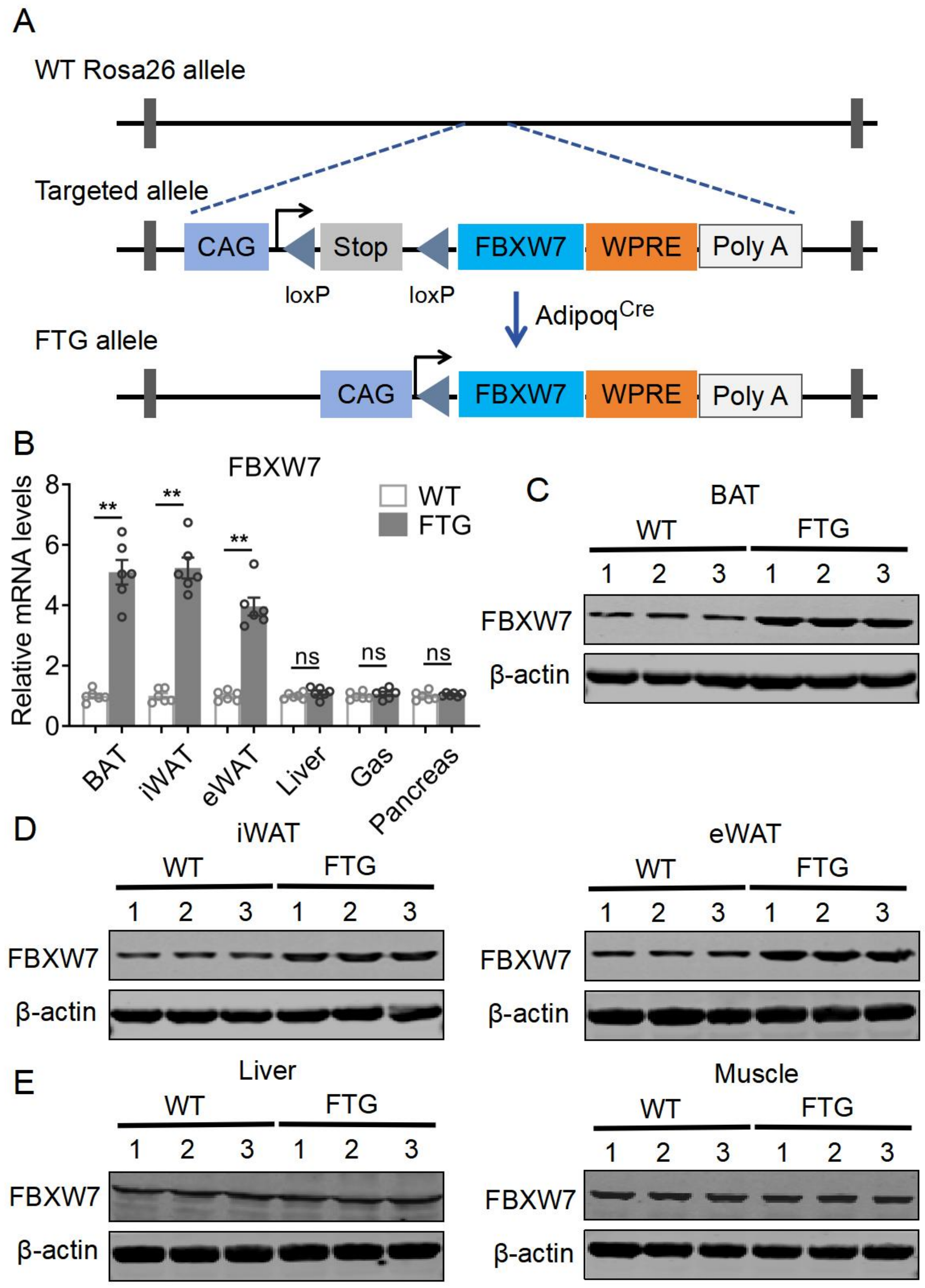

Appendix Figure S2. Construction of FBXW7-FTG mouse.

(A)Targeting strategy for adipocyte-specific overexpression of FBXW7.

(B)Relative mRNA levels of Fbxw7 in WT and FBXW7-FTG mice, n=6.

(C-E) Representative protein levels of FBXW7 in BAT (C), iWAT, eWAT (D), liver and muscle (E) of WT and FBXW7-FTG mice.

Data information: All data are representative of three individual experiments. n refers to biological replicates. (B) Data are presented as mean ± SEM, unpaired two-tailed Student's t-test, **p<0.01. ns: not significant.

Appendix Figure S3

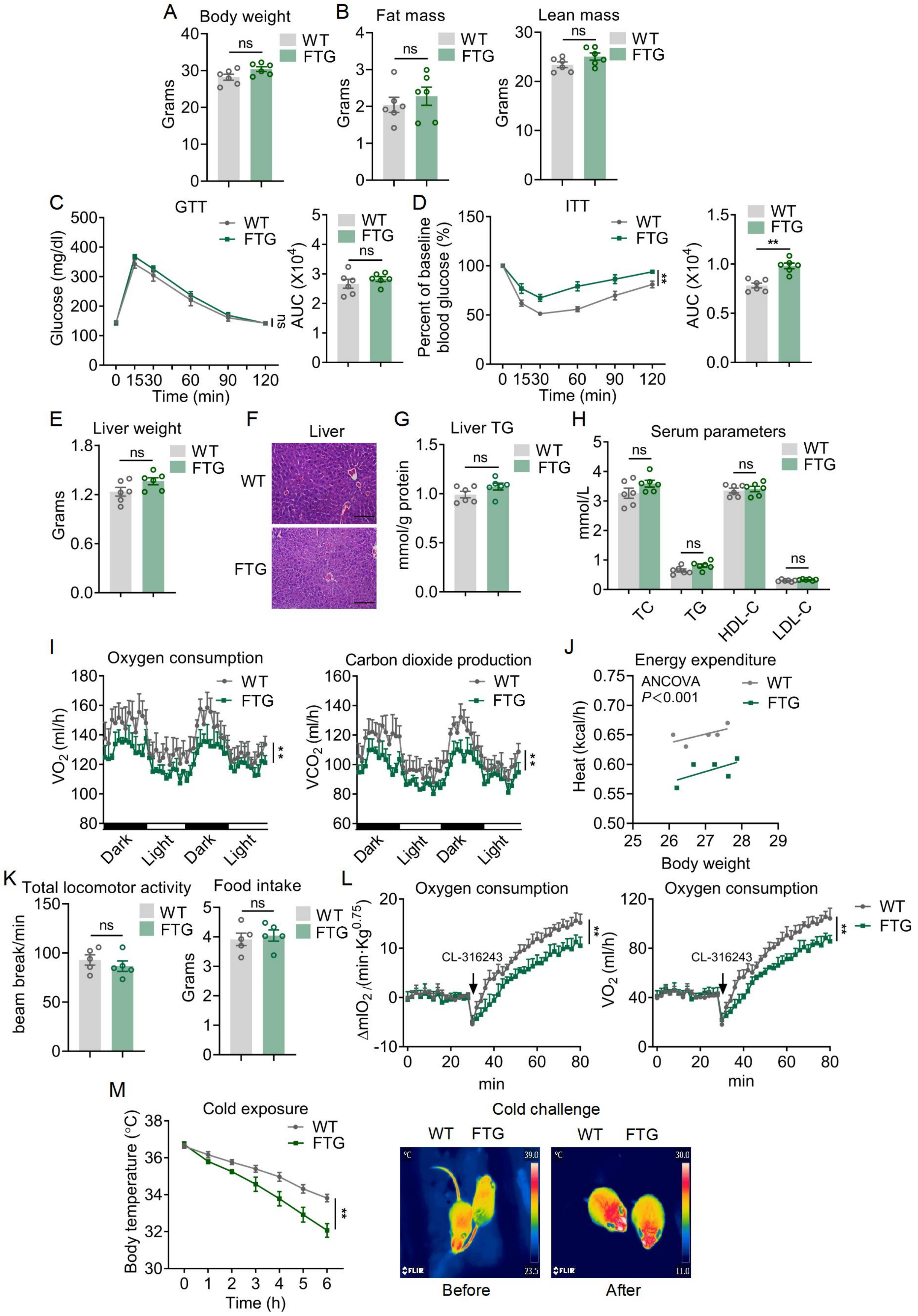

Appendix Figure S3. Basic metabolic performances of WT and FBXW7-FTG mice.

(A-M) Metabolic performances of chow diet fed WT and FBXW7-FTG mice.

(A) Body weight, n=6; (B) Fat mass and lean mass, n=6; (C) Glucose tolerance test (GTT) and area under the curve (AUC), n=6; (D) Insulin tolerance test (ITT) and AUC, n=6; (E) Liver weights, n=6; (F) Representative H&E staining of liver, scale bar represents 100 μm; (G) Liver triglycerides level, n=6; (H) Serum parameters, n=6; (I, J) Energy expenditure as shown by oxygen consumption, carbon dioxide production and heat at an ambient temperature of 22°C, n=5; (K) Total locomotor activity and food intake, n=6; (L) CL-316243 induced VO2 of mice anesthetized at 30°C (Left: ml of ΔO2·min-1·(kg of body weight-0.75); Right: ml of O2·hour-1), n=5; (M) Rectal temperatures and infrared thermography of mice during 6 h cold exposure at 4°C, n=6.

Data information: All data are representative of three individual experiments. n refers to biological replicates. (A, B, E, G, H, K) Data are presented as mean ± SEM, unpaired two-tailed Student's t-test, ns: not significant. (C, D, I, L, M) Data are presented as mean ± SEM. Two-way ANOVA followed by Sidak's multiple comparison test was conducted and statistical significance denoted as *p<0.05 and **p<0.01, ns: not significant. (D: p<0.0001; I: Left panel: p<0.0001; I: Right panel: p<0.0001; L: Left panel: p<0.0001; L: Right panel: p<0.0001; M: p<0.0001). (J) Data are presented as mean ± SEM. ANCOVA was used to analyze energy expenditure by SPSS software, p<0.001.

Appendix Figure S4

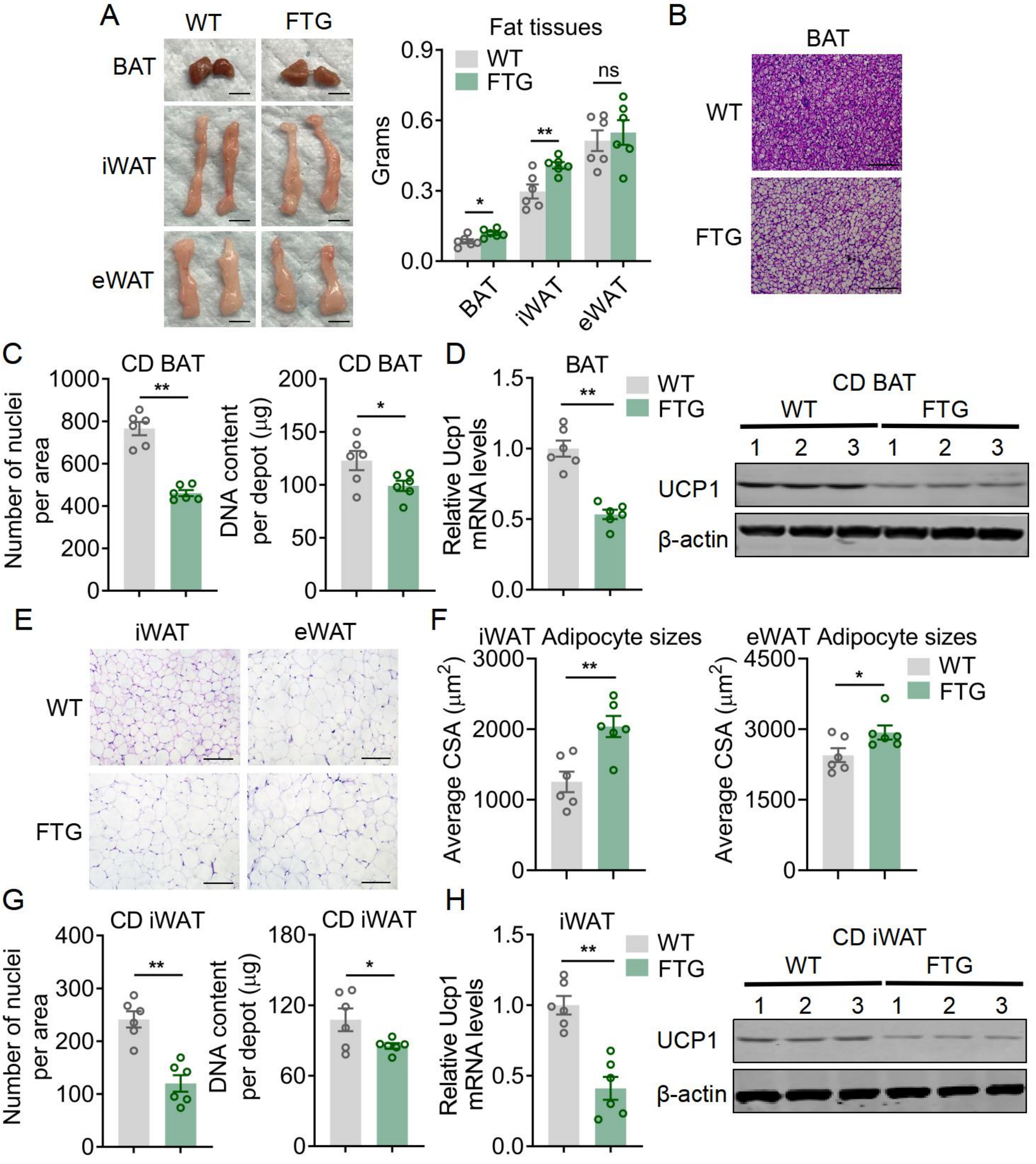

Appendix Figure S4. Adipose-specific overexpression of FBXW7 in mice reduces brown fat programs.

(A-H) Phenotypic characteristics of chow diet fed WT and FBXW7-FTG mice.

(A) Tissue weights of brown (BAT), inguinal (iWAT) and epididymal (eWAT) fat pads, scale bar represents 5 mm, n=6; (B) Representative images of H&E staining of BAT, scale bar represents 100 µm; (C) Nuclei densities and genomic DNA content in BAT, n=6; (D) Relative mRNA and protein level of Ucp1 in BAT, n=6; (E) Representative images of H&E staining of iWAT and eWAT, scale bar represents 100 µm; (F) Quantitative analysis of adipocyte sizes and CSA frequency distribution of iWAT and eWAT, n=6; (G) Nuclei densities and genomic DNA content in iWAT, n=6; (H) Relative mRNA and protein level of Ucp1 in iWAT, n=6.

Data information: All data are representative of three individual experiments. n refers to biological replicates. (A-H) Data are presented as mean ± SEM, unpaired two-tailed Student's t-test, *p<0.05, **p<0.01, ns: not significant.

Appendix Figure S5

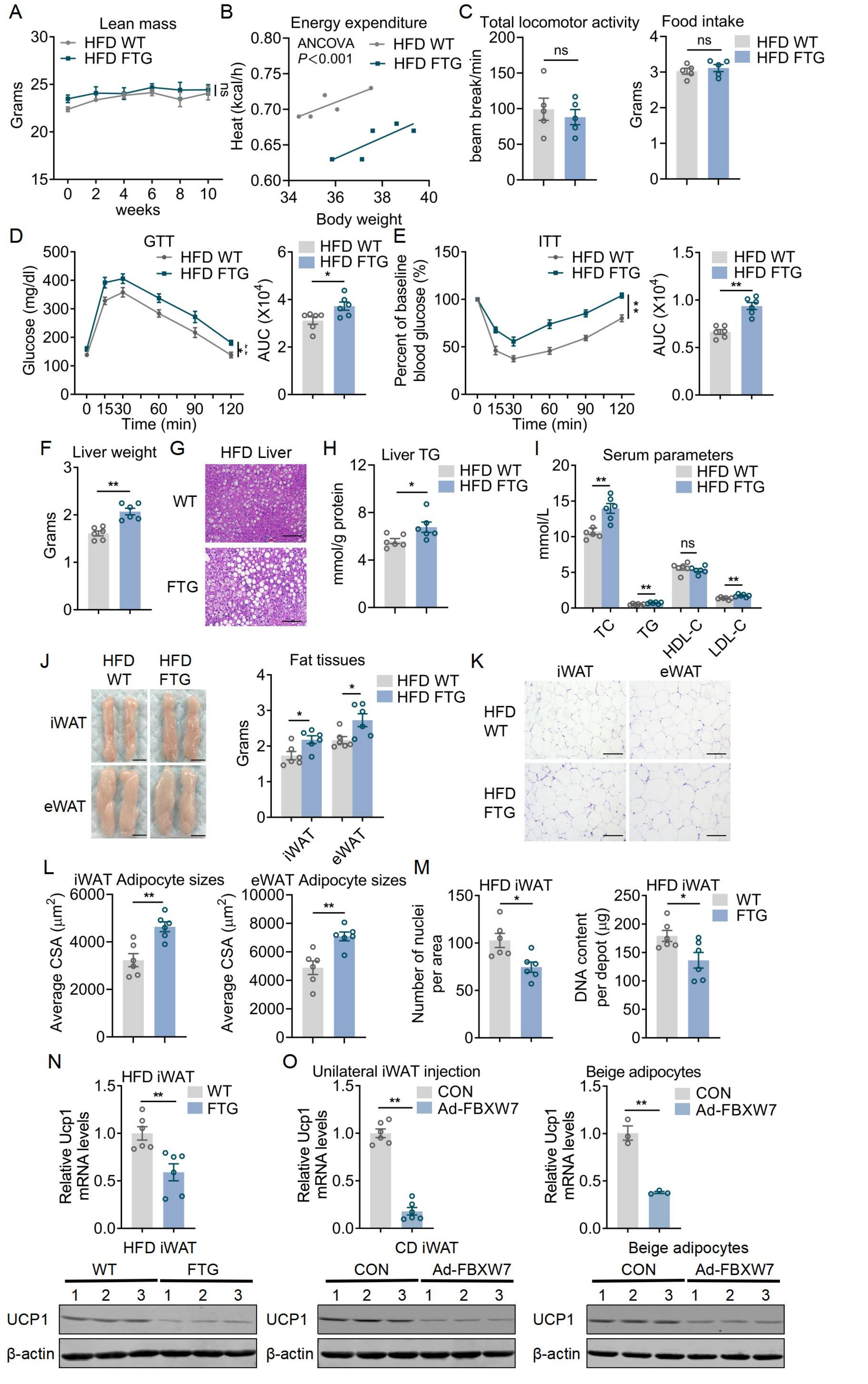

Appendix Figure S5. Basic metabolic performances of WT and FBXW7-FTG mice under HFD.

(A-O) Metabolic performances of high fat diet fed WT and FBXW7-FTG mice.

(A) Lean mass, n=6; (B) Energy expenditure, n=5; (C) Total locomotor activity and food intake, n=5; (D) Glucose tolerance test (GTT) and AUC; (E) Insulin tolerance test (ITT) and AUC, n=6; (F) Liver weight, n=6; (G) Representative H&E staining of liver, scale bar represents 100 µm; (H) Liver triglycerides levels, n=6; (I) Serum parameters, n=6; (J) Weights of iWAT and eWAT, scale bar represents 1 cm, n=6; (K) Representative images of H&E staining of iWAT and eWAT, scale bar represents 100 µm; (L) Quantitative analysis of adipocyte sizes and CSA frequency distribution of iWAT and eWAT, n=6; (M) Nuclei densities and genomic DNA content in iWAT, n=6; (N) Relative mRNA and protein level of Ucp1 in iWAT, n=6; (O) in inguinal fat pads injected with ADV-GFP or ADV-FBXW7 for 4 days, n=6 and in beige adipocytes infected with ADV-GFP or ADV- FBXW7 for 48h, n=3.

Data information: All data are representative of three individual experiments. n refers to biological replicates. (A, D, E) Data are presented as mean ± SEM. Two-way ANOVA followed by Sidak's multiple comparison test was conducted and statistical significance denoted as *p<0.05, **p<0.01, ns: not significant. (D: p=0.0224; E: p<0.0001). (B) Data are presented as mean ± SEM. ANCOVA was used to analyze energy expenditure by SPSS software, p<0.001. (C, F, H-J, L-O) Data are presented as mean ± SEM, unpaired two-tailed Student's t-test, *p<0.05, **p<0.01, ns: not significant.

Appendix Figure S6

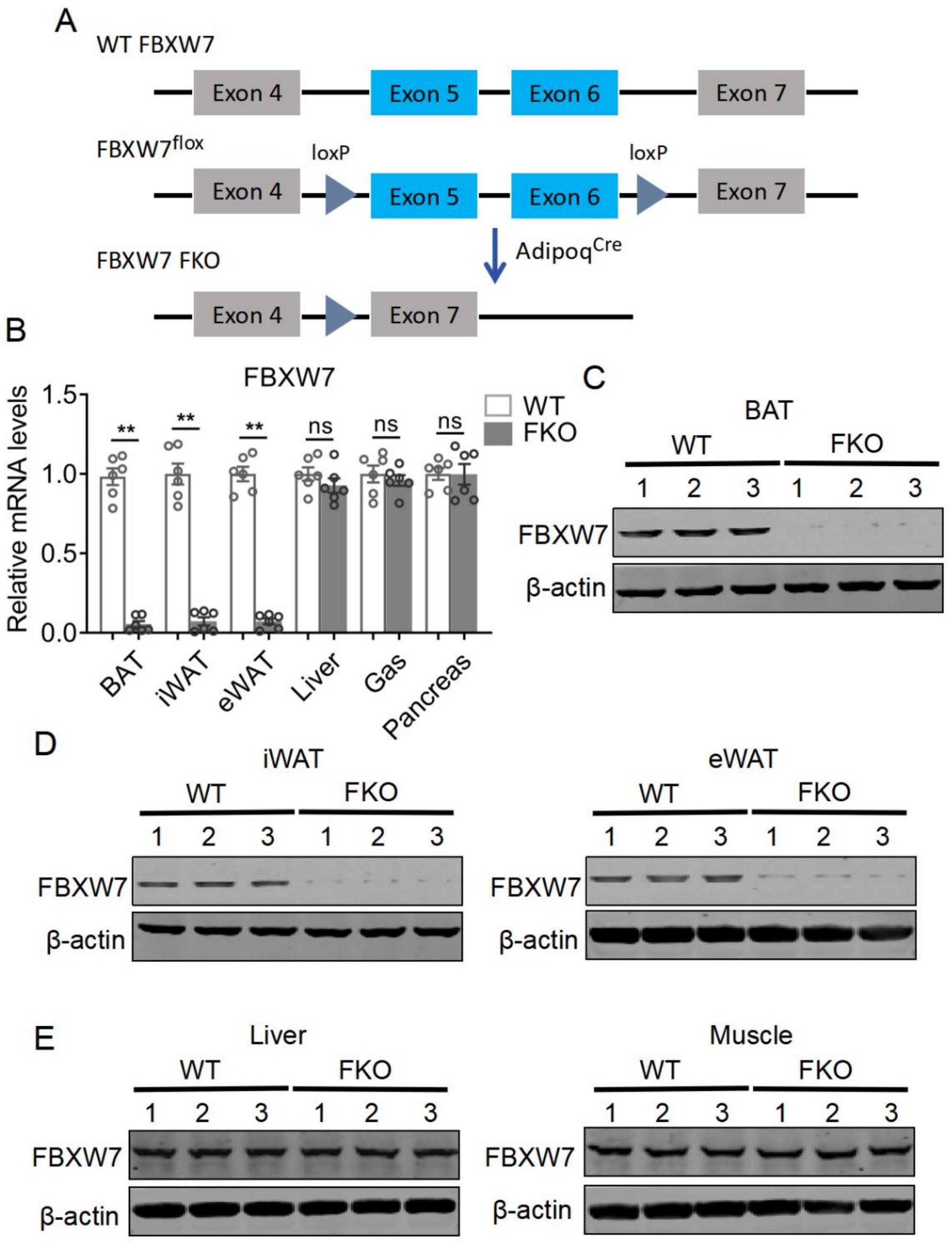

Appendix Figure S6. Construction of FBXW7-FKO mouse.

(A)Targeting strategy for adipocyte-specific knockout of FBXW7.

(B)Relative mRNA levels of Fbxw7 in WT and FBXW7-FKO mice, n=6.

(C-E) Representative protein levels of FBXW7 in BAT (C), iWAT, eWAT (D), liver and muscle (E) of WT and FBXW7-FKO mice

Data information: All data are representative of three individual experiments. n refers to biological replicates. (B) Data are presented as mean ± SEM, unpaired two-tailed Student's t-test, **p<0.01. ns: not significant.

Appendix Figure S7

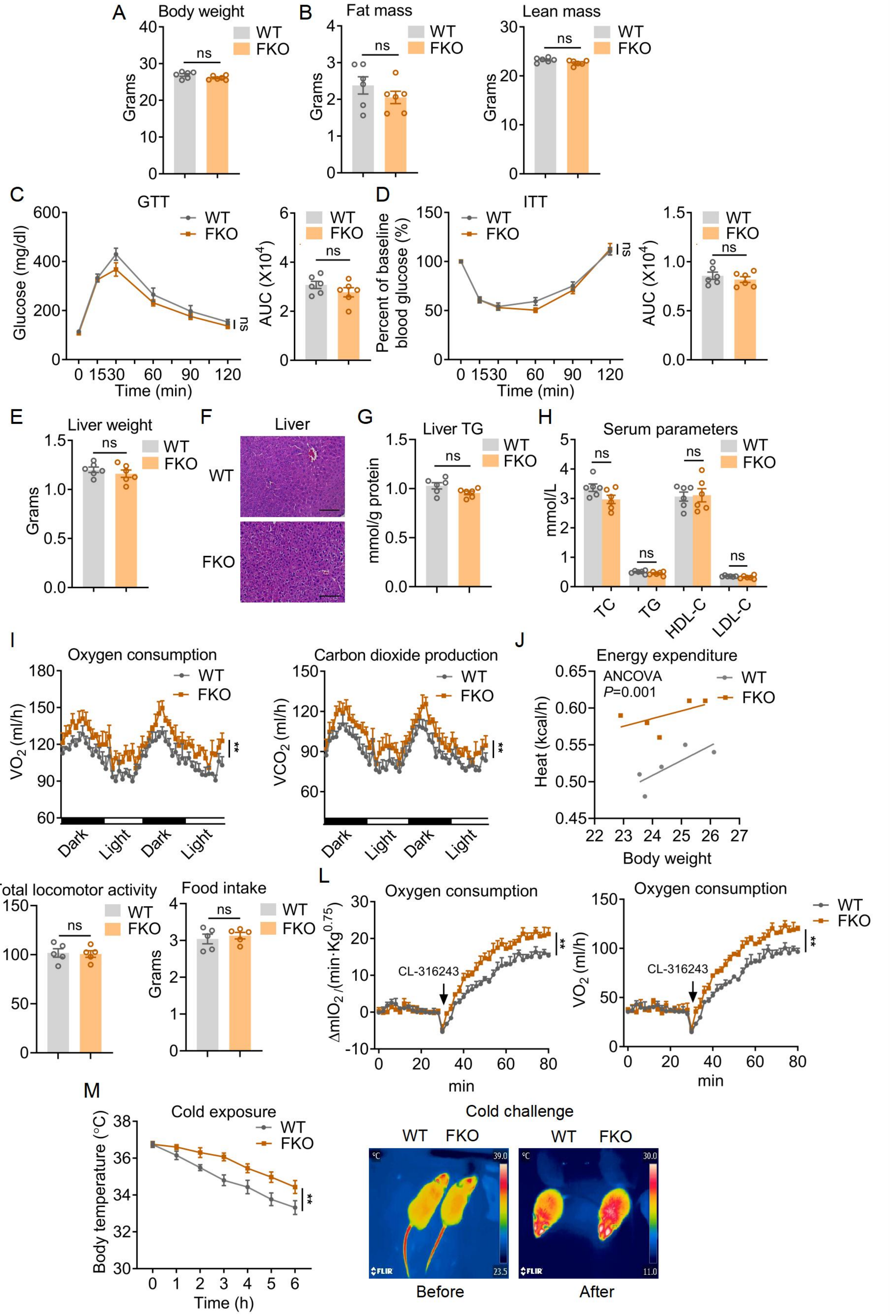

Appendix Figure S7. Basic metabolic performances of WT and FBXW7-FKO mice.

(A-M) Metabolic performances of chow diet fed WT and FBXW7-FKO mice.

(A, B) Body weight, fat mass and lean mass, n=6; (C, D) Glucose tolerance test (GTT) and AUC and Insulin tolerance test (ITT) and AUC, n=6; (E) Liver weight, n=6; (F) Representative H&E staining of liver, scale bar represents 100 μm; (G) Liver triglycerides levels, n=6; (H) Serum parameters, n=6; (I,J) Energy expenditure as shown by oxygen consumption, carbon dioxide production and heat of mice at an ambient temperature of 22°C, n=5; (K) Total locomotor activity and Food intake, n=5; (L) CL-316243 induced VO2 of mice anesthetized at 30°C (Left: ml of ΔO2·min-1·(kg of body weight-0.75); Right: ml of O2·hour-1), n=5; (M) Rectal temperatures and infrared thermography of mice during 6 h cold exposure at 4°C, n=6.

Data information: All data are representative of three individual experiments. n refers to biological replicates. (A, B, E, G, H, K) Data are presented as mean ± SEM, unpaired two-tailed Student's t-test, ns: not significant. (C, D, I, L, M) Data are presented as mean ± SEM. Two-way ANOVA followed by Sidak's multiple comparison test was conducted and statistical significance denoted as *p<0.05 and **p<0.01, ns: not significant. (I: Left panel: p<0.0001; I: Right panel: p<0.0001; L: Left panel: p<0.0001; L: Right panel: p<0.0001; M: p<0.0001). (J) Data are presented as mean ± SEM, **p<0.01. ANCOVA was used to analyze energy expenditure by SPSS software, p=0.001.

Appendix Figure S8

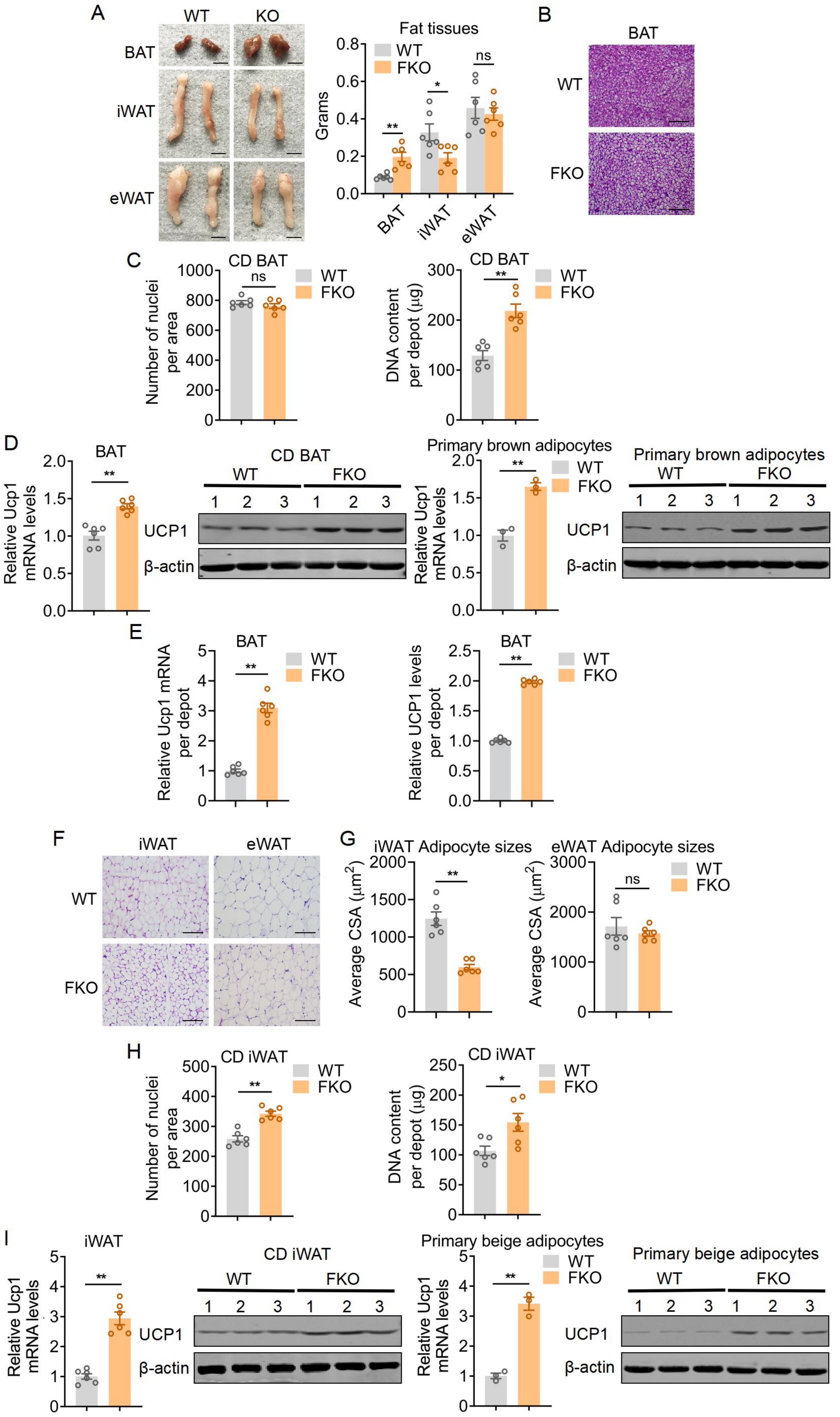

Appendix Figure S8. Adipose-specific ablation of FBXW7 mice promotes energy metabolism.

(A-I) Phenotypic characteristics of chow diet fed WT and FBXW7-FKO mice.

(A) Tissue weights of brown (BAT), inguinal (iWAT) and epididymal (eWAT) fat pads, scale bar represents 5 mm, n=6; (B) Representative images of H&E staining of BAT, iWAT and eWAT, scale bar represents 100 µm; (C) Nuclei densities and genomic DNA content in BAT, n=6; (D) Relative mRNA and protein level of UCP1 from WT and FBXW7-FKO mice including in BAT, n=6 and differentiated brown adipocytes from BAT SVF, n=3; (E) Relative Ucp1 mRNA and protein levels per depot of BAT, n=6; (F) Representative images of H&E staining of iWAT and eWAT, scale bar represents 100 µm; (G) Quantitative analysis of adipocyte sizes and CSA frequency distribution of iWAT and eWAT, n=6; (H) Nuclei densities and genomic DNA content in iWAT, n=6; (I) Relative mRNA and protein level of Ucp1 from WT and FBXW7-FKO mice including in iWAT, n=6 and differentiated beige adipocytes from iWAT SVF, n=3.

Data information: All data are representative of three individual experiments. n refers to biological replicates. (A, C, D, E, G, H, I) Data are presented as mean ± SEM, unpaired two-tailed Student's t-test, **$p<0.01$. ns: not significant.

Appendix Figure S9

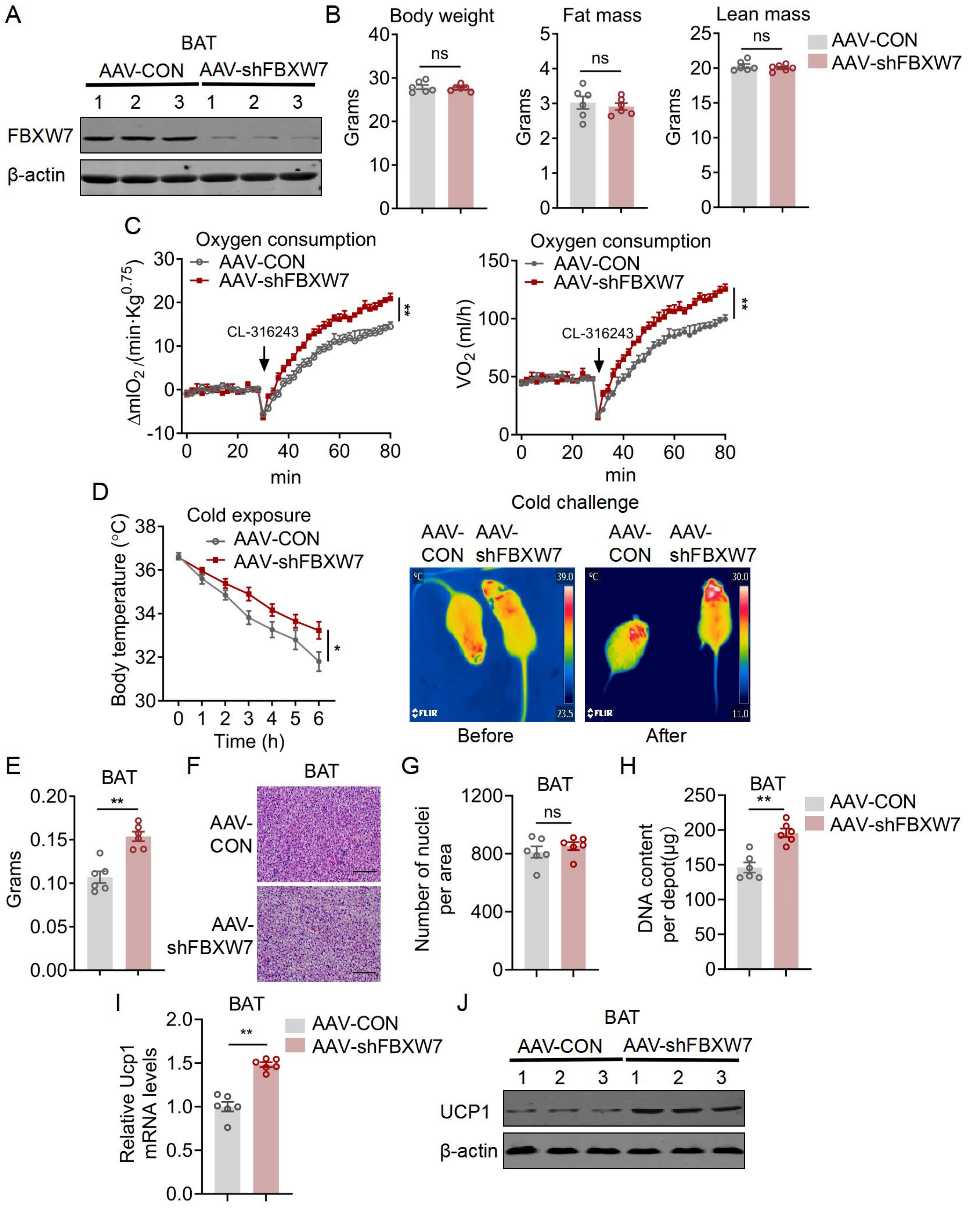

Appendix Figure S9. FBXW7 knockdown in mice led to brown fat expansion and increased energy expenditure under HFD.

(A-J) Metabolic analysis of high fat diet fed AAV-CON and AAV-shFBXW7 mice. AAV-shFBXW7 and AAV-CON were injected in BAT of indicated mice and mice were fed with HFD.

(A) Western blot analysis of FBXW7 protein levels in BAT; (B) Body weight, fat mass and lean mass, n=6; (C) CL-316243 induced VO2 of mice anesthetized at 30°C (Left: ml of ΔO2·min-1·(kg of body weight-0.75); Right: ml of O2·hour-1), n=5; (D) Rectal temperatures and infrared thermography of mice during 6 h cold exposure at 4°C, n=6; (E) Weights of BAT, n=6; (F) Representative images of H&E staining of BAT, scale bar represents 100 μm; (G) Nuclei densities, n=6 and (H) genomic DNA content in BAT, n=6; (I) Relative mRNA and (J) protein level of Ucp1 in BAT, n=6.

Data information: All data are representative of three individual experiments. n refers to biological replicates. (B, E, G, H, I) Data are presented as mean ± SEM, unpaired two-tailed Student's t-test, $**p<0.01$, ns: not significant. (C, D) Data are presented as mean ± SEM. Two-way ANOVA followed by Sidak's multiple comparison test was conducted and statistical significance denoted as $*p<0.05$, $**p<0.01$. (C: Left panel: $p<0.0001$; C: Right panel: $p<0.0001$; D: $p=0.0321$).

Appendix Figure S10

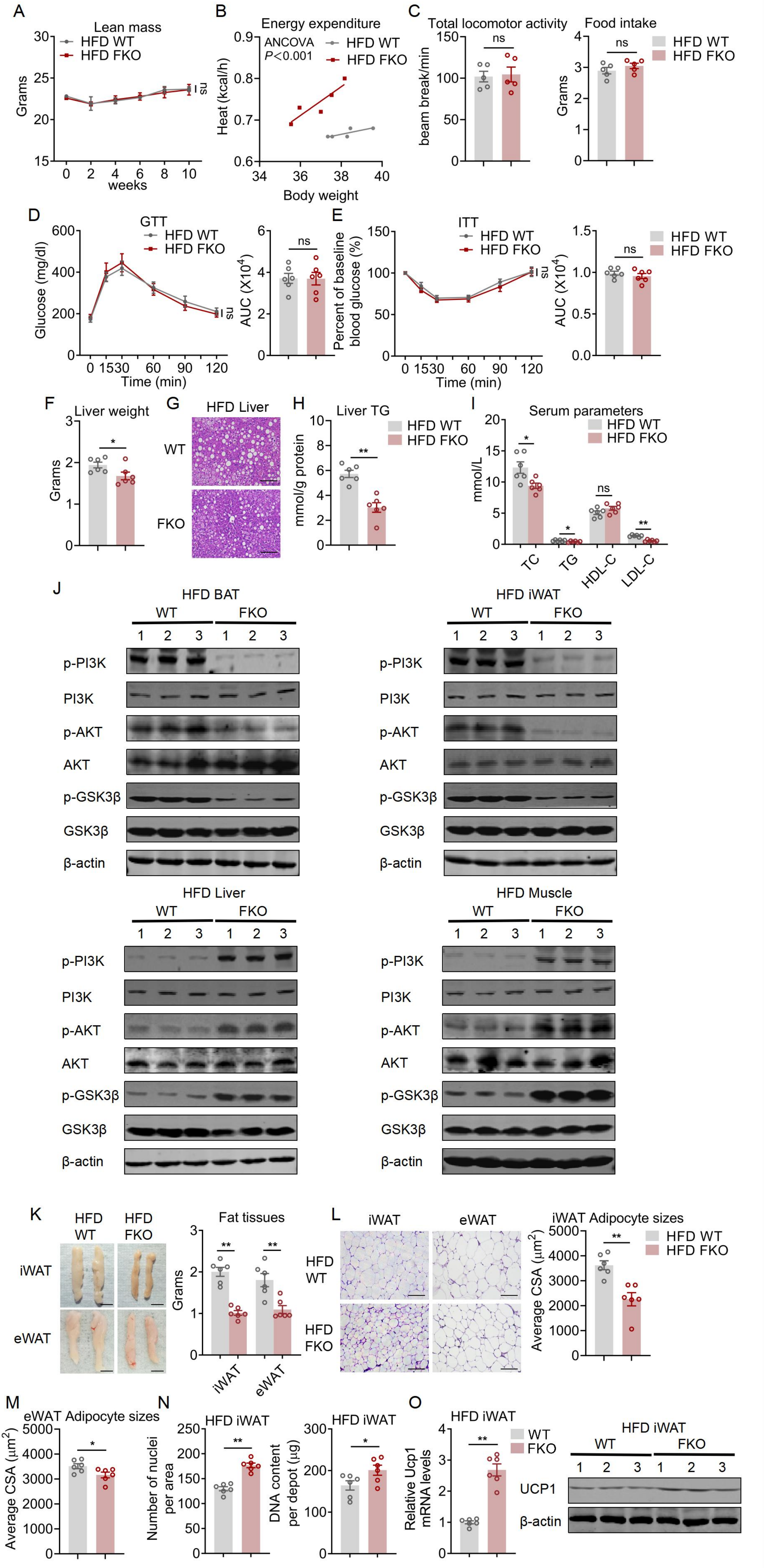

Appendix Figure S10. Basic metabolic performances of WT and FBXW7-FKO mice under HFD.

(A-O) Metabolic performances of high fat diet fed WT and FBXW7-FKO mice.

(A)Lean mass, n=6; (B) Energy expenditure, n=5; (C) Total locomotor activity and food intake, n=5; (D) Glucose tolerance test (GTT) and AUC, n=6; (E) Insulin tolerance test (ITT) and AUC, n=6; (F) Liver weight, n=6; (G) Representative H&E staining of liver, scale bar represents 100 µm; (H) Liver triglycerides levels, n=6; (I) Serum parameters, n=6; (J) Relative protein level of p-PI3K, PI3K, p-AKT, AKT, p-GSK3β and GSK3β of BAT, iWAT, liver and muscle; (K) Weights of iWAT and eWAT, scale bar represents 1 cm, n=6; (L) Representative images of H&E staining of iWAT and eWAT, scale bar represents 100 µm; (M) Quantitative analysis of adipocyte sizes and CSA frequency distribution of iWAT and eWAT, n=6; (N) Nuclei densities and genomic DNA content in iWAT, n=6; (O) Relative mRNA and protein level of Ucp1 in iWAT of HFD WT and FBXW7-FKO mice, n=6.

Data information: All data are representative of three individual experiments. n refers to biological replicates. (A, D, E) Data are presented as mean ± SEM. Two-way ANOVA followed by Sidak's multiple comparison test was conducted and statistical significance denoted as ns: not significant. (B) Data are presented as mean ± SEM. ANCOVA was used to analyze energy expenditure by SPSS software, $p<0.001$. (C, F, H, I, K-O) Data are presented as mean ± SEM, unpaired two-tailed Student's t-test, $^{*}p<0.05$, $^{**}p<0.01$. ns: not significant.

Appendix Figure S11

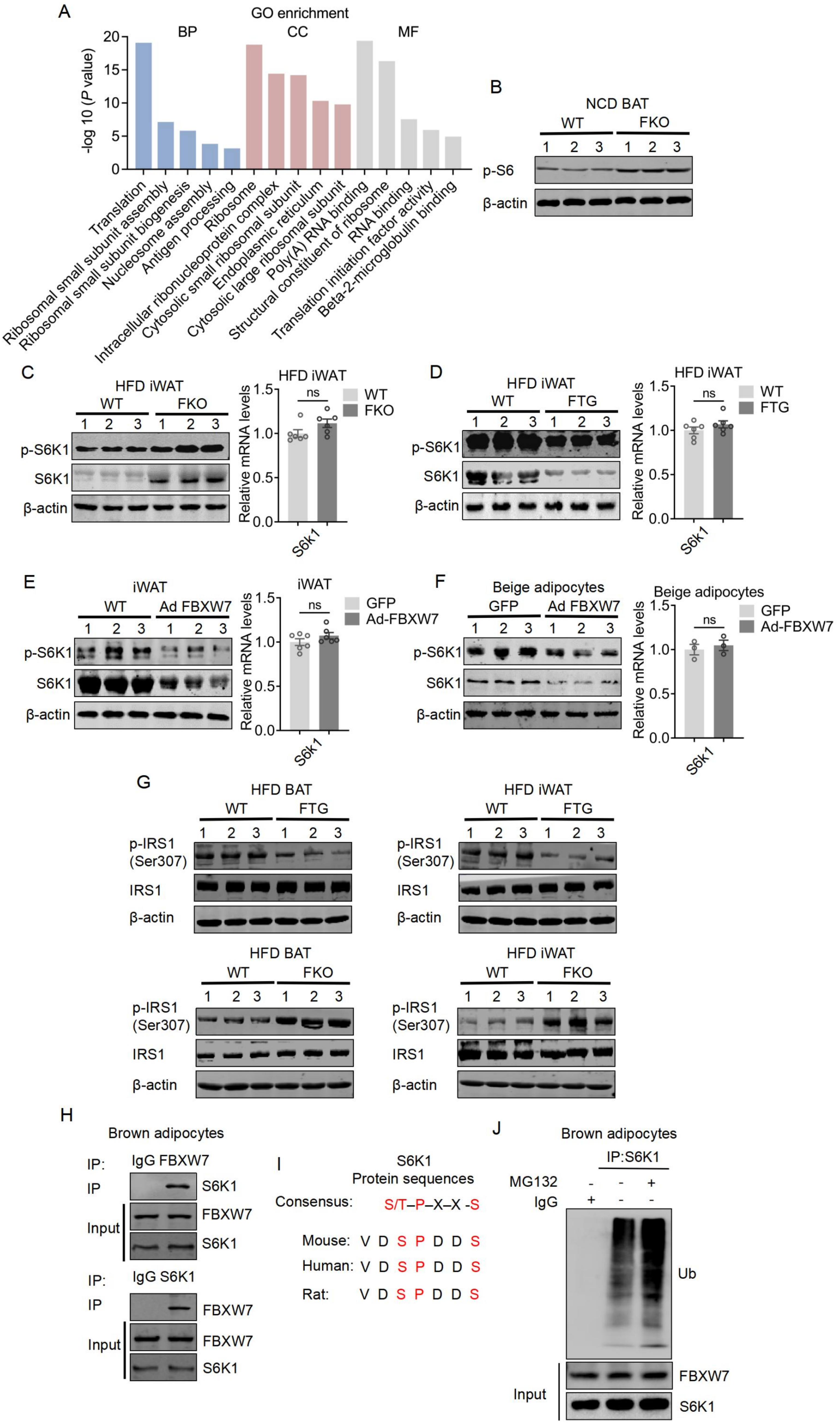

Appendix Figure S11. FBXW7 deficiency in fat enhanced p-S6K1 and S6K1 protein level.

(A) GO enrichment analysis of BAT protein in WT and FBXW7-FKO mice fed with high fat.

(B) Relative protein of p-S6 in BAT of normal chow diet fed WT and FBXW7-FKO mice.

(C-F) p-S6K1 and S6K1 protein levels, as well as relative mRNA levels of S6K1 in iWAT from WT, FBXW7-FKO or FBXW7-FTG mice fed with high fat (C, D), n=6; in inguinal fat pads injected with ADV-GFP or ADV-FBXW7 for 4 days (E), n=6 and in beige adipocytes infected with ADV-GFP or ADV- FBXW7 for 48h (F), n=3.

(G)p-IRS1(Ser307) and IRS1 protein levels in BAT and iWAT from WT, FBXW7-FTG or FBXW7-FKO mice fed with high fat.

(H)Immunoprecipitation of FBXW7 and S6K1 in brown adipocytes.

(I) Amino acid sequence alignment of S6K1 from various species, highlighting the conserved FBXW7 recognition sequence S/T-P-X-X-S.

(J) Ubiquitination assay of S6K1 in brown adipocytes with or without the proteasome inhibitor MG132.

Data information: All data are representative of three individual experiments. n refers to biological replicates. (A) GO enrichment analysis was performed with the R package clusterProfiler, with a Bonferroni correction and an adjusted p-value of 0.05. (C–F), Data are presented as mean ± SEM, unpaired two-tailed Student's t-test, ns: not significant.

Appendix Figure S12

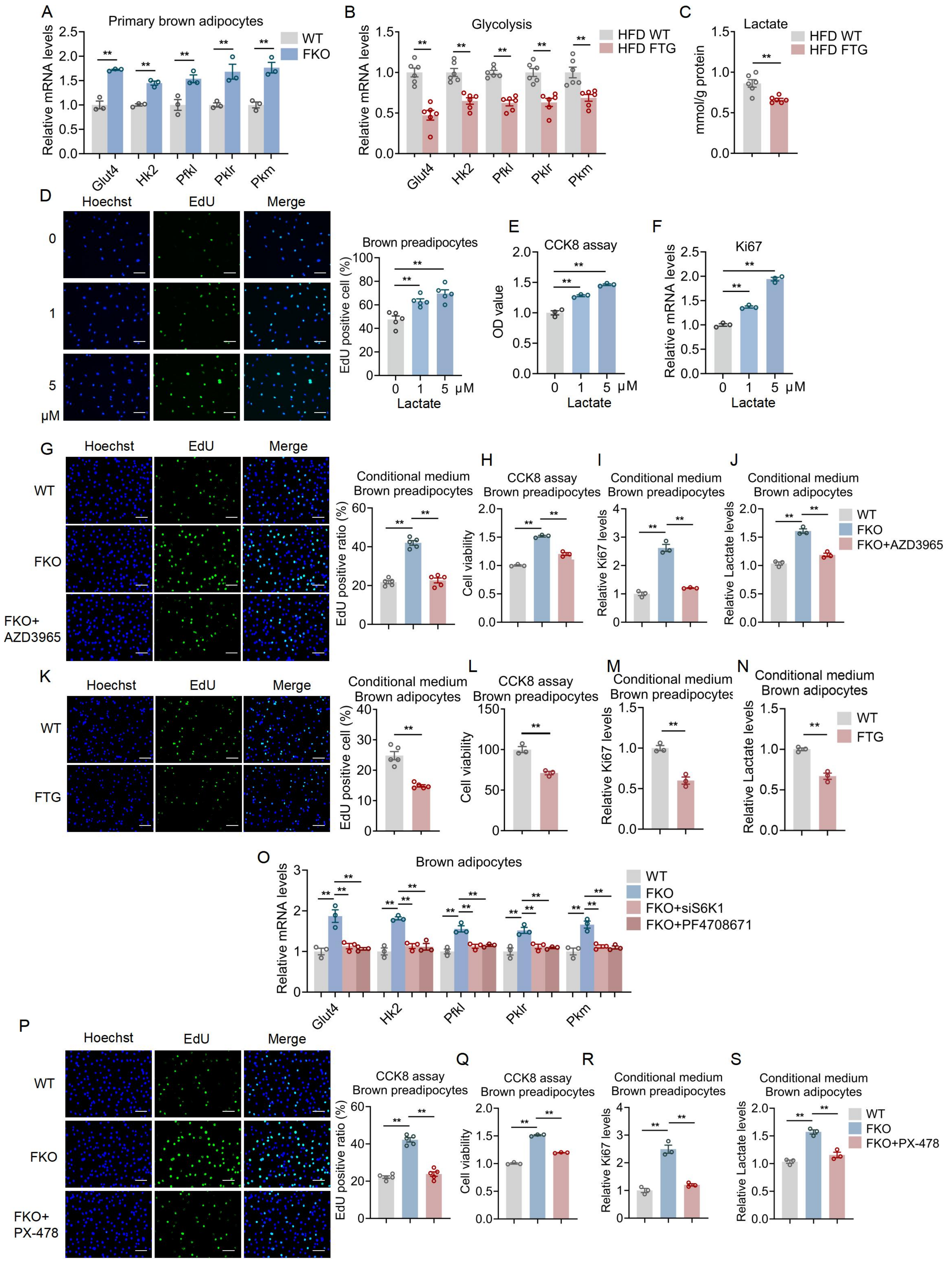

Appendix Figure S12. FBXW7 modulation in fat affects lactate production, which mediates brown preadipocyte proliferation.

(A) Relative mRNA level of glycolytic genes in differentiated brown adipocytes from SVF in WT and FBXW7-FKO mice, n=3.

(B, C) Relative mRNA level of glycolytic genes and lactate levels in BAT from WT and FBXW7-FTG mice fed with high fat diet, n=6.

(D-F) Representative images of EdU staining (D), scale bar represents 50 μm, n=5, cell viability (E) , n=3 and mRNA level of Ki67 (F) of brown preadipocytes treated with lactate at 1, 5 and 10 μM for 24h, n=3.

(G-J) EdU staining, scale bar represents 50 μm, n=5; (H) cell viability, n=3 and (I) mRNA level of Ki67 of brown preadipocytes treated with the culture medium of differentiated brown adipocytes from SVF in WT and FBXW7-FKO mice pretreated with lactate transporter inhibitor AZD3965 at 1 μM, n=3; (J) Relative lactate level in the culture medium of differentiated brown adipocytes from SVF in BAT of WT and FBXW7-FKO mice pretreated with AZD3965 at 1 μM, n=3.

(K-N) EdU staining, scale bar represents 50 μm, n=5; (L) cell viability, n=3 and (M) mRNA level of Ki67 of brown preadipocytes treated with the culture medium of differentiated brown adipocytes from SVF in WT and FBXW7-FTG mice, n=3; (N) Relative lactate level in the culture medium of differentiated brown adipocytes from SVF in BAT of WT and FBXW7-FTG mice, n=3.

(O) Relative mRNA level of glycolytic genes in differentiated brown adipocytes from SVF in WT and FBXW7-FKO mice pretreated with S6K1 siRNA or S6K1 inhibitor-PF4708671 at 10 μM, n=3.

(P-S) EdU staining, scale bar represents 50 μm, n=5; (Q) cell viability, n=3 and (R) mRNA level of Ki67 of brown preadipocytes treated with the culture medium of differentiated brown adipocytes from SVF in WT and FBXW7-FKO mice pretreated with Hif-1α inhibitor-PX-478 at 10 μM, n=3; (S) Relative lactate level in the culture medium of differentiated brown adipocytes from SVF in BAT of WT and FBXW7-FKO mice pretreated with Hif-1α inhibitor-PX-478 at 10 μM, n=3.

Data information: All data are representative of three individual experiments. n refers to biological replicates. (A-C, K-N) Data are presented as mean ± SEM, unpaired two-tailed Student's t-test, $^{**}p<0.01$. (D-J, O-S), Data are presented as mean ± SEM. One-way ANOVA followed by Dunnett's multiple comparison test was conducted and statistical significance denoted as $^{**}p<0.01$. (D: $p=0.003$; E: $p<0.0001$; F: $p<0.0001$; G: $p<0.0001$; H: $p<0.0001$; I: $p<0.0001$; J: $p<0.0001$; O: $p<0.01$; P: $p<0.0001$; Q: $p<0.0001$; R: $p<0.0001$; S: $p<0.0001$).

Appendix Figure S13

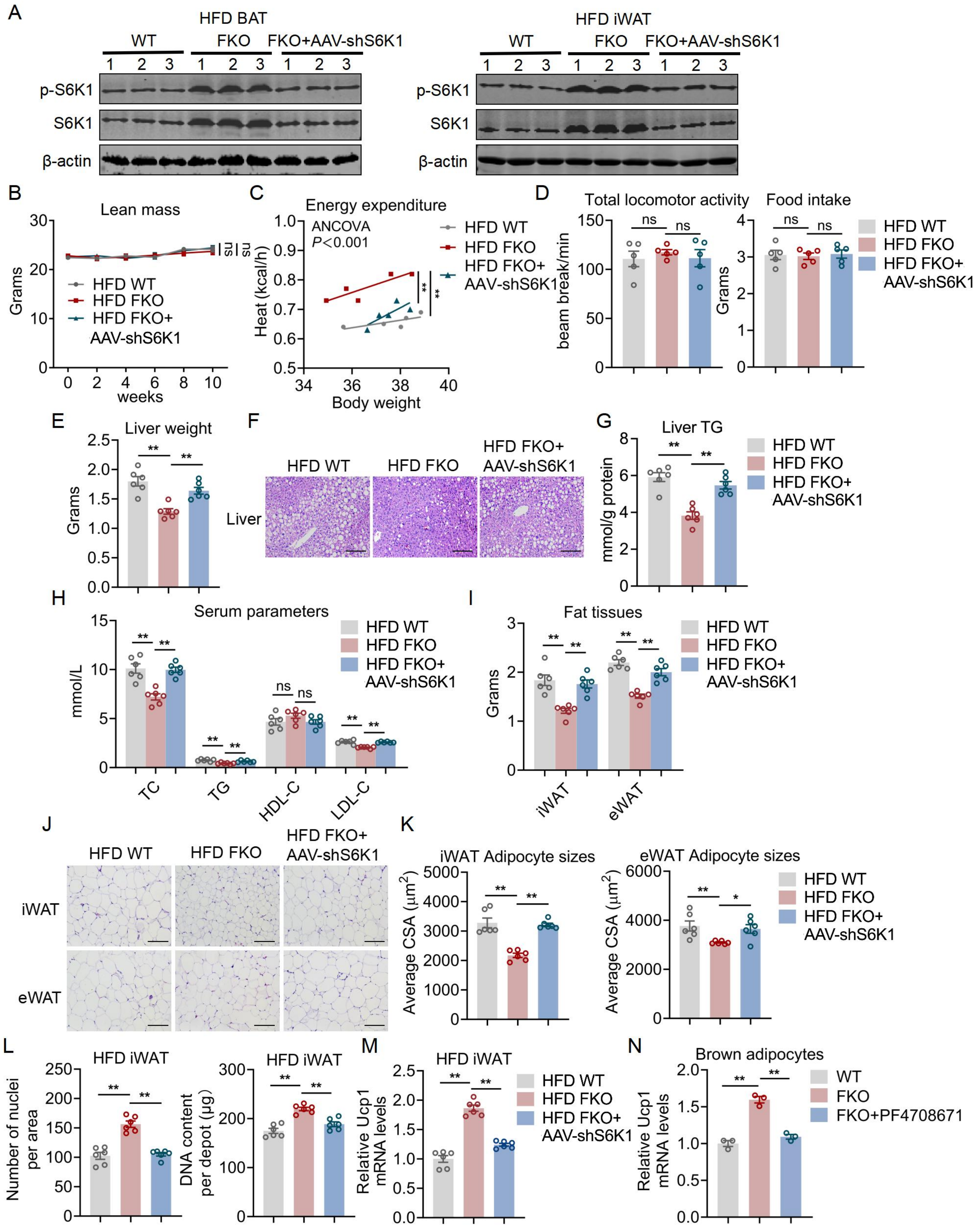

Appendix Figure S13. S6K1 acts downstream of FBXW7 for the regulation of metabolic performances.

(A-N) Metabolic analysis of high fat diet fed WT, FBXW7-FKO mice and FBXW7 FKO+AAV-shS6K1 mice. AAV-shS6K1 and control AAV were injected both in BAT and iWAT of indicated mice and mice were fed with HFD.

(A) Western blot analysis of pho-S6K1 and S6K1 protein levels in BAT and iWAT; (B) Energy expenditure, n=5; (C) Lean mass, n=6; (D) Total locomotor activity and food intake, n=5; (E) Liver weight, n=6; (F) Representative H&E staining of liver, scale bar represents 100 μm; (G) Liver triglycerides levels, n=6; (H) Serum parameters, n=6; (I) Weights of iWAT and eWAT, n=6; (J) Representative images of H&E staining of iWAT and eWAT, scale bar represents 100 μm; (K) Quantitative analysis of adipocyte sizes and CSA frequency distribution of iWAT and eWAT, n=6; (L) Nuclei densities and genomic DNA content in iWAT, n=6; (M) Relative mRNA level of Ucp1 in iWAT, n=6; (N) Relative mRNA level of Ucp1 in brown adipocytes from SVF in BAT of WT and FBXW7-FKO mice pretreated with S6K1 inhibitor-PF4708671 at 10 μM, n=3.

Data information: All data are representative of three individual experiments. n refers to biological replicates. (B) Data are presented as mean ± SEM. Two-way ANOVA followed by Tukey's multiple comparison test was conducted and statistical significance denoted as ns: not significant. (B) Data are presented as mean ± SEM. ANCOVA was used to analyze energy expenditure by SPSS software, $p<0.001$. (D–N) Data are presented as mean ± SEM. One-way ANOVA followed by Dunnett's multiple comparison test was conducted and statistical significance denoted as *$p<0.05$, **$p<0.01$, ns: not significant. (E: $p=0.0001$; G: $p<0.0001$; H: TC: $p<0.0001$; TG; $p=0.0002$; LDL-C: $p<0.0001$; I: iWAT: $p=0.0001$; eWAT: $p<0.0001$; K: Left panel: $p<0.0001$; right panel: $p=0.0196$; L: Left panel: $p<0.0001$; right panel: $p<0.0001$; M: $p<0.0001$; N: $p<0.0001$).

Appendix Figure S14

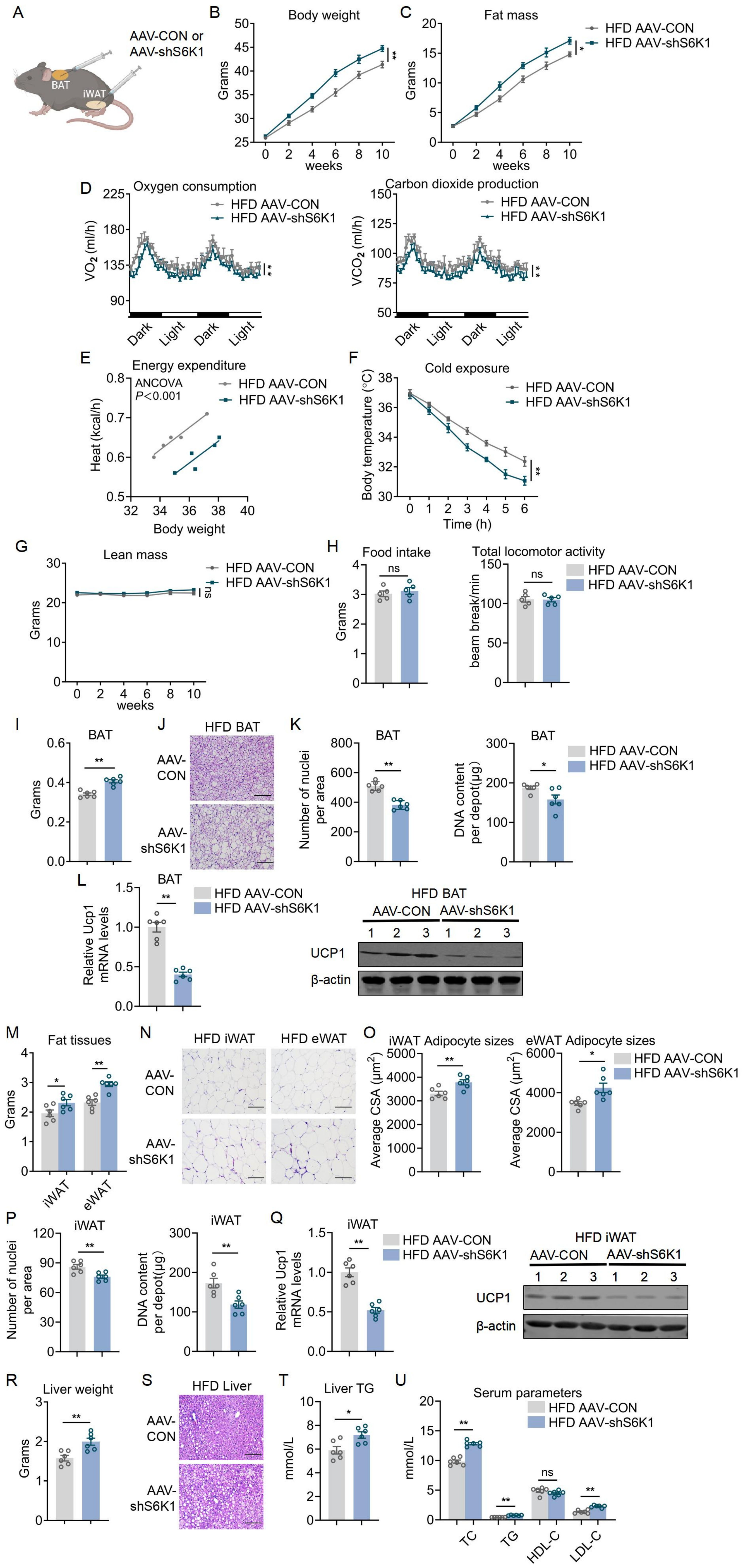

Appendix Figure S14. S6K1 knockdown in BAT and iWAT regulated thermogenic fat function and energy metabolism.

(A-U) Metabolic performances of high fat diet fed AAV-CON and AAV-shS6K1 mice. AAV-shS6K1 and AAV-CON were injected both in BAT and iWAT of indicated mice and mice were fed with HFD.

(A-T) Experimental model for knocking down S6K1 in BAT and iWAT; (B) Body weight, n=6; (C) Fat mass, n=6; (D) Energy expenditure, n=5; (E) CL-316243 induced VO2 of mice anesthetized at 30°C (Left: ml of ΔO2·min-1·(kg of body weight-0.75); Right: ml of O2·hour-1), n=5; (F) Rectal temperatures and infrared thermography of mice during 6 h cold exposure at 4°C, n=6; (G) Lean mass, n=6; (H) Food intake and total locomotor activity, n=5; (I) Weights of BAT, n=6; (J) Representative images of H&E staining of BAT, scale bar represents 100 μm; (K) Nuclei densities and genomic DNA content in BAT, n=6; (L) Relative mRNA and protein level of Ucp1 in BAT, n=6; (M) Weights of iWAT and eWAT, n=6; (N) Representative images of H&E staining of iWAT and eWAT, scale bar represents 100 μm; (O) Quantitative analysis of adipocyte sizes and CSA frequency distribution of iWAT and eWAT, n=6; (P) Nuclei densities and genomic DNA content in iWAT, n=6; (Q) Relative mRNA and protein level of Ucp1 in iWAT, n=6; (R) Weight of Liver, n=6; (S) Representative H&E staining of liver, scale bar represents 100 μm; (T) Liver triglycerides levels, n=6; (U) Serum parameters of AAV-CON and AAV-shS6K1 mice, n=6.

Data information: All data are representative of three individual experiments. n refers to biological replicates. (B, C, D, F, G) Data are presented as mean ± SEM. Two-way ANOVA followed by Sidak's multiple comparison test was conducted and statistical significance denoted as *p<0.05, **p<0.01, ns: not significant. (B: p=0.0029; C: p=0.0112; D: Left panel: p<0.0001; D: Right panel: p<0.0001; F: p<0.0001). (E) Data are presented as mean ± SEM. ANCOVA was used to analyze energy expenditure by SPSS software, p<0.001. (G–T) Data are presented as mean ± SEM, unpaired two-tailed Student's t-test, *p<0.05, **p<0.01, ns: not significant.

Appendix Table S1. The metabolic traits of subjects carrying different rs1351903 genotypes

	AA (n=1969)	AG (n=4093)	GG (n=2335)
Age (year)	56.21±1.3	56.44±9.97	56.22±5.33
Sex (male%)	41.34%	41.97%	40.99%
Body mass index (kg/m^2)	24.67±2.41	24.66±1.02	24.76±5.31
HbA1c (%(mmol/mol))	5.55±0.25	5.54±0.2	5.54±0.35
Glycated albumin (mmol/L)	13.67±2.26	13.66±0.94	13.66±2.08
Uric acid (µmol/L)	301.6±119.5	303.3±263	301.62±11.5
Free fatty acids (µmol/L)	522.73±22.5	536.65±84	535.33±10.5

Data are shown as mean±SEM or N%. AA, wild-type; AG, heterozygous; GG, mutation.

Appendix Table S2. PCR primers used in the study

Gene name	Primer sequence (5′→3′)	
m36B4	Forward	AGATTCGGGATATGCTGTTGGC
	Reverse	TCGGGTCCTAGACCAGTGTTC
mUcp1	Forward	GGCCCTTGTAAACAACAAAATAC
	Reverse	GGCAACAAGAGCTGACAGTAAAT
mS6k1	Forward	AAATGCTGCTTCTC GTCTTG
	Reverse	CTTGGAATCAAACTGACTCACATC
mFbxw7	Forward	ACCGCTTCTTCCTCAGTTCC
	Reverse	GCCAGCTTGCTACTTCTTGG
mKi67	Forward	GCCATAACCCGAAAGAGCAG
	Reverse	CCAGTTTACGCTTTGCAGGT
mFbxw7α	Forward	CTCACCAGCTCTCCTCTCCATT
	Reverse	GCTGAACATGGTACAAGGCCA
mFbxw7β	Forward	TTGTCAGAGACTGCCAAGCAG
	Reverse	GACTTTGCATGGTTTCTTTCCC
mFbxw7γ	Forward	AACCATGGCTTGGTTCCTGTTG
	Reverse	CAGAACCATGGTCCAACTTTC
ROSA-GT	Forward	AGTCGCTCTGAGTTGTTATCAG
	Reverse	TGAGCATGTCTTTAATCTACCTCGATG
ROSA26-Test(L)-R3	Reverse	GTCAATGGAAAGTCCCTATTGGCGT
FBXW7-GT	Forward	ATTGATACAAACTGGAGACGAGG
	Reverse	ATAGTAATCCTCCTGCCTTGGC
Cre700	Forward	GCCGCATTACCGGTCGATGCAAGA
	Reverse	GTGGCAGATGGCGCGGCAACACCATT
mFbxw7-cds	Forward	ATGAATCAGGAACTGCTCTC
	Reverse	TCATTTCATGTCCACATCAA
mS6k1-cds	Forward	ATGAGGCGACGACGGAGGCG
	Reverse	TCATAGATTCATCCGCAGGT
